# A Review on Biodentine, a Contemporary Dentine Replacement and Repair Material

**DOI:** 10.1155/2014/160951

**Published:** 2014-06-16

**Authors:** Özlem Malkondu, Meriç Karapinar Kazandağ, Ender Kazazoğlu

**Affiliations:** ^1^Department of Prosthodontics, Faculty of Dentistry, Yeditepe University, Bagdat Caddesi 238, Goztepe, 34728 Istanbul, Turkey; ^2^Department of Endodontics, Faculty of Dentistry, Yeditepe University, Bagdat Caddesi 238, Goztepe, 34728 Istanbul, Turkey

## Abstract

Biodentine is a calcium-silicate based material that has drawn attention in recent years and has been advocated to be used in various clinical applications, such as root perforations, apexification, resorptions, retrograde fillings, pulp capping procedures, and dentine replacement. There has been considerable research performed on this material since its launching; however, there is scarce number of review articles that collates information and data obtained from these studies. Therefore, this review article was prepared to provide the reader with a general picture regarding the findings about various characteristics of the material. The results of a PubMed search were classified and presented along with some critical comments where necessary. The review initially focuses on various physical properties of the material with subheadings and continues with biocompatibility. Another section includes the review of studies on Biodentine as a vital pulp treatment material and the article is finalized with the summary of some case reports where the material has been used.

## 1. Background

Calcium silicate based materials have gained popularity in recent years due to their resemblance to mineral trioxide aggregate (MTA) and their applicability in cases where MTA is indicated. Although various calcium silicate based products have been launched to the market recently, one of these has especially been the focus of attention and the topic of a variety of investigations. This material is the “Biodentine” calcium silicate based product which became commercially available in 2009 (Septodont, http://www.septodontusa.com/) and that was specifically designed as a “dentine replacement” material. Biodentine has a wide range of applications including endodontic repair (root perforations, apexification, resorptive lesions, and retrograde filling material in endodontic surgery) and pulp capping and can be used as a dentine replacement material in restorative dentistry. The material is actually formulated using the MTA-based cement technology and the improvement of some properties of these types of cements, such as physical qualities and handling [1]. Since “Biodentine” has frequently been pronounced in recent literature and serves as an important representative of tricalcium silicate based cements, a review of the studies pertaining to its properties will be contributory in generating a clearer picture regarding the general characteristics of this frequently acknowledged material. This review article makes a general analysis, provides a summary of studies on Biodentine, and critically evaluates the existing knowledge regarding the properties of the product. A search was conducted in PubMed by inserting keywords “Biodentine,” “dentistry,” and “endodontic repair.” Articles were retrieved that were published since the launching of the material into the market and classified according to the topic that they focused on. A total of 52 papers were included that consisted of those directly focusing on Biodentine as well as relavant papers that do not include Biodentine but are related to dental materials in general.

## 2. Physical Properties of Biodentine

### 2.1. Composition

The product file of Biodentine states that the powder component of the material consists of tricalcium silicate, dicalcium silicate, calcium carbonate and oxide filler, iron oxide shade, and zirconium oxide. Tricalcium silicate and dicalcium silicate are indicated as main and second core materials, respectively, whereas zirconium oxide serves as a radiopacifier. The liquid, on the other hand, contains calcium chloride as an accelerator and a hydrosoluble polymer that serves as a water reducing agent. It has also been stated that fast setting time, one unique characteristics of the product, is achieved by increasing particle size, adding calcium chloride to the liquid component, and decreasing the liquid content. The setting period of the material is as short as 9–12 minutes. This shorter setting time is an improvement compared to other calcium silicate materials [1]. Some authors have indicated that there are few studies on the properties of newly developed materials such as Biodentine [2]. The material is characterized by the release of calcium when in solution [3, 4]. Tricalcium silicate based materials are also defined as a source of hydroxyapatite when they are in contact with synthetic tissue fluid [5–7].

A search of the literature reveals a few studies that aim to further investigate the composition and setting characteristics of the material. Grech et al. [7] assessed the composition of materials and leachate of a prototype cement of tricalcium silicate and radiopacifier (without any additives) and 2 commercially available tricalcium silicate cements, one of which was Biodentine. Their main purpose was to assess the effect of the additives used in commercial brands. The authors characterized the hydrated cements using scanning electron microscopy (SEM) and X-ray energy dispersive analysis (EDX), X-ray diffraction (XRD), and Fourier transform infrared spectroscopy (FT-IR). They concluded that Biodentine resulted in the formation of calcium silicate hydrate and calcium and hydroxide, leached in solution. The materials, when hydrated, consisted of a cementitious phase, rich in calcium, silicone, and a radiopacifying material. Biodentine was further described as having calcium carbonate in powder and the carbonate phase of the material was verified by XRD and FT-IR analysis. The Biodentine powder also had inclusions of calcium carbonate which were relatively large compared to cement particles. There were hydration products around the circumference of the calcium carbonate particles. The authors added that calcium carbonate acts as a nucleation site, enhancing the microstructure [7].

Similar results were reported by Camilleri et al. [8] who compared the composition of Biodentine and MTA Angelus with experimentally produced laboratory cement consisting of tricalcium silicate and zirconium oxide. Their analysis also showed that tricalcium silicate was the main constituent of Biodentine and no dicalcium silicate or calcium oxide was detected. They further noted that Biodentine consisted of other additives for the enhancement of the material. Calcium carbonate was used as 15% in the powder component [8].

An important feature of the calcium carbonate additive was to act as a nucleation site for C–S–H, thereby reducing the duration of the induction period, leading to a faster setting time. The tricalcium silicate grains in Biodentine were also reported to be finer and calcium chloride and a water soluble polymer were included in the liquid portion [8].

### 2.2. Setting Time

Grech et al. [9] investigated the setting time of Biodentine using an indentation technique while the material was immersed in Hank's solution. The authors described that this methodology uses a Vicat apparatus with a needle of specific mass. The setting time of the mixture is calculated as the time taken from the start of mixing until the indentor fails to leave a mark on the set material surface.

The setting time of Biodentine was determined as 45 minutes. This short setting time was attributed to the addition of calcium chloride to the mixing liquid [9]. Calcium chloride has also been shown to result in accelerated setting time for mineral trioxide aggregate [10]. An interesting finding of the study by Grech et al [9] was that the highest setting period was determined for Bioaggregate, another tricalcium silicate based material. The product sheet of Biodentine [1] indicates the setting time as 9 to 12 minutes, which is shorter than the one observed in the study by Grech et al. [9]. However, 9–12 minutes indicated in the product sheet is the initial setting time, whereas Grech et al. [9] evaluated the final setting time. Therefore, both papers are not comparable.

Villat et al. [11] preferred a different methodology for the assessment of the setting time, the impedance spectroscopy that assesses the changes in electrical resistivity. Interestingly, impedance values were stabilized after 5 days for the glass ionomer cement while at least 14 days were necessary for the calcium silicate based cement. The authors speculated that this result was due to the higher porosity for Biodentine cement, characterizing higher capacity of ion exchanges between the material and its environment [11].

### 2.3. Compressive Strength

Compressive strength is considered as one of the main physical characteristics of hydraulic cements. Considering that a significant area of usage of products such as Biodentine is vital pulp therapies, it is essential that the cement has the capacity to withstand masticatory forces, in other words, sufficient compressive strength to resist external impacts [2].

The product sheet of Biodentine states that a specific feature of Biodentine is its capacity to continue improving in terms of compressive strength with time until reaching a similar range with natural dentine [1]. In the study by Grech et al. [9], Biodentine showed the highest compressive strength compared to the other tested materials. The authors attributed this result to the enhanced strength due to the low water/cement ratio used in Biodentine. They stated that this mode of the material is permissible as a water soluble polymer is added to the mixing liquid. Kayahan et al. [2] evaluated the compressive strength from another perspective and drew conclusions specifically pertaining to clinical usage. Considering that acid etching is one of the steps following the application of Biodentine for the provision of mechanical adhesion, the authors aimed to assess whether any alterations exist in terms of compressive strength following the etching procedure. They concluded that acid etching procedures after 7 days did not reduce the compressive strength of ProRoot MTA and Biodentine [2]. Although these studies are limited and further research is definitely warranted; they hold promise for Biodentine as a suitable material for use in procedures, such as vital pulp therapies, where there is direct exposure to external masticatory forces and compressive strength capacity is of primary significance. Furthermore, in a study by Koubi et al. [12], Biodentine was used as a posterior restoration and revealed favorable surface properties such as good marginal adaptation until 6 months.

### 2.4. Microhardness

Grech et al. [9] evaluated the microhardness of the material using a diamond shaped indenter. Their results showed that Biodentine displayed superior values compared to Bioaggregate and IRM. Camilleri [13], in a study comparing the physical properties of Biodentine with a conventional glass ionomer (Fuji IX) and a resin modified glass ionomer (Vitrebond), showed that Biodentine exhibited higher surface microhardness compared to the other materials when unetched. On the other hand, there was no difference in the microhardness of different materials when they were etched [13].

### 2.5. Bond Strength

Considering that Biodentine is recommended for use as a dentine substitute under permanent restorations, studies were performed that assess the bond strength of the material with different bonding systems. Odabaş et al. [14] evaluated the shear bond strength of an etch-and-rinse adhesive, a 2-step self-etch adhesive and a 1-step self-etch adhesive system to Biodentine at different intervals. No significant differences were found between all of the adhesive groups at the same time intervals (12 minutes and 24 hours). When different time intervals were compared, the lowest bonding value was obtained for the etch-and-rinse adhesive at a 12-minute period, whereas the highest was obtained for the 2-step self-etch adhesive at the 24-hour period [14].

Another area of use of Biodentine, specifically from an endodontic point of view, is the repair of perforations, which is likely to be encountered in clinical practice. It is essential that a perforation repair material should have sufficient amount of push-out bond strength with dentinal walls for the prevention of dislodgement from the repair site. Aggarwal et al. [15] studied the push-out bond strengths of Biodentine, ProRoot MTA, and MTA Plus in furcal perforation repairs. Push-out bond strength increased with time. Their results showed that the 24 h push-out strength of MTA was less than that of Biodentine and blood contamination affected the push-out bond strength of MTA Plus irrespective of the setting time. A favorable feature of Biodentine determined by the authors was that blood contamination had no effect on the push-out bond strength, irrespective of the duration of setting time [15].

El-Ma'aita et al. [16] aimed to assess the effect of smear layer on the push-out bond strength of calcium silicate cements and whether the removal of this layer would have any overall influence on the bonding characteristics of these materials. The authors used Biodentine, ProRoot MTA, and Harvard MTA as root fillings. The results showed that the removal of the smear layer significantly reduced the push-out bond strengths of calcium silicate cements and the smear layer was a critical issue that determines the bond strength between dentine and calcium silicate cements such as Biodentine. The authors attributed this result to the inability of calcium silicate cement particles to penetrate the dentinal tubules due to their particle size. They speculated that the smear layer is important in the formation of the interfacial layer and may be involved in the mineral interaction between the CSC and radicular dentin. It is appropriate to mention that it is not customary to use calcium silicate based materials for the obturation of the entire root canal system and such an approach might not be preferable especially in narrow and curved root canals. On the other hand, the study by El-Ma'aita et al. [16] is significant because it successfully demonstrated the bonding characteristics of these popular materials which are unique in contemporary dental applications.

Hashem et al. [17], in a recently published report, drew attention to another issue in terms of bond strength characteristics of Biodentine with overlying materials that was not mentioned previously. Biodentine is a weak restorative material in its early setting phase. The authors advocated that, in case of a laminate/layered definitive restoration, the placement of the overlying resin composite must be delayed for more than 2 weeks so that Biodentine material will undergo adequate maturation to withstand contraction forces from the resin composite [17].

In a study by Guneser et al. [18], Biodentine showed considerable performance as a repair material even after being exposed to various endodontic irrigation solutions, such as NaOCl, chlorhexidine, and saline, whereas MTA had the lowest push-out bond strength to root dentin.

### 2.6. Porosity and Material-Dentine Interface Analysis

Tricalcium silicate based materials are especially indicated in cases such as perforation repair, vital pulp treatments, and retrograde fillings where a hermetic sealing is mandatory. Therefore, the degree of porosity plays a very important role in the overall success of treatments performed using these materials, because it is critical factor that determines the amount of leakage. Porosity has been shown to have an impact upon numerous other factors including adsorption, permeability, strength, and density. It has further been stated that the maximum pore diameter, which corresponds to the largest leak in the sample, along with bacterial size and their metabolites, will be indicative of the leakage that occurs along the root-end filling materials [19]. Camilleri et al. [20] evaluated the root dentine to material interface of Bioaggregate, Biodentine, a prototype radiopacified tricalcium silicate cement (TCS-20-Zr) and intermediate restorative material (IRM) when used as root-end filling materials in extracted human teeth after 28 days of dry storage and immersion in HBSS using a confocal microscope together with fluorescent tracers and also a field emission gun scanning electron microscope. They used a prototype material (TCS-20-Zr) similar to Biodentine in composition which was composed of a cementitious phase, namely, tricalcium silicate and a radiopacifier (zirconium oxide) with no additives. The reason for testing such a prototype material was to assess the effects that the additives in Biodentine have on the porosity of the material and to detect any changes in the material characteristics at the root-dentine to material interface. The testing was performed in two environmental conditions, namely, dry or immersed in a physiological solution. According to their results, Biodentine and IRM exhibited the lowest level or degree of porosity. The confocal microscopy used in conjunction with fluorescent tracers demonstrated that dry storage resulted in gaps at the root dentine to material interface and also cracks in the material and Biodentine was the most affected one from ambient conditions. Dry storage of Biodentine caused changes in the material microstructure and cracks at the root dentine to Biodentine interface. Furthermore, the gaps occurring due to material shrinkage allowed the passage of the fluorescent microspheres. These gaps were defined as significant as they had the potential to allow the ingress and transmission of microorganisms [20].

The authors' results were significant from a clinical standpoint because it can be interpreted from the results of the study that the type of treatment performed is a critical factor that determines the porosity and subsequent leakage occurring thereafter. In case the procedure is a retrograde filling where there is a continuously moist environment, lesser porosity that occurs by Biodentine is advantageous. However, in procedures such as liners, bases, or dentine replacement, the material is generally kept dry which might pose a problem in terms of porosity and result in the formation of gaps at the interface, leading to bacterial passage. This leads to the conclusion that caution must be exercised during the selection of Biodentine in certain clinical conditions where moisture is not necessarily present.

Another study on porosity was one by De Souza et al. [21] where Biodentine was compared to other silicate based cements, IRoot BP Plus, Ceramicrete, and ProRoot MTA using micro—CT characterization. The authors indicated that no significant difference in porosity between IRoot BP PlusVR, BiodentineVR, and Ceramicrete were observed. In addition, no significant differences were found in porosity between the new calcium silicate-containing repair cements and the gold-standard MTA. The authors made similar conclusions in terms of the behavior of tricalcium based materials and drew similarity between them and the conventional MTA in terms of microleakage, solubility, and microfractures in the clinical setting [21].

Gjorgievska et al. [22] conducted a study where the interfacial properties of 3 different bioactive dental substitutes were compared, one of which was Biodentine. Whilst the cavity adaptation of bioglass was poor owing to its particle size, both glass ionomers and calcium silicate cements yielded favorable results as dentine substitutes. During SEM analysis, Biodentine crystals appeared firmly attached to the underlying dentine surface. The authors further emphasized the resemblance of the interfacial layer formed between Biodentine and dentine to the hard tissue layer formed by ProRoot MTA and further drew attention to the hydroxyapatite crystal growth. Also, the Biodentine crystals appeared to be firmly attached to the underlying dentine surface. Although they found no evidence of ionic exchange, they concluded that the excellent adaptability of this material to the underlying dentine is dependent on mainly micromechanical adhesion [22].

Atmeh et al. [23] studied the interfacial properties of Biodentine and glass ionomer cement by different microscopy and spectroscopy methods and determined the existence of interfacial tag-like structures along the dentine. The alkaline caustic effect of hydration products degraded the collagenous component of dentine next to Biodentine. This altered dentine structure was only observed beneath the Biodentine samples.

### 2.7. Radiopacity

Radiopacity is an important property expected from a retrograde or repair material as these materials are generally applied in low thicknesses and they need to be easily discerned from surrounding tissues. The ISO 6876:2001 has established 3 mm Al as the minimum radiopacity value for endodontic cements [24]. Meanwhile, according to ANSI/ADA specification number 57, all endodontic sealers should be at least 2 mm Al more radiopaque than dentin or bone [25]. For the determination of the radiopacities of filling materials, the method developed by Tagger and Katz [26] is generally used where radiographic images of the material are taken alongside an aluminium step-wedge.

Zirconium oxide is used as a radiopacifier in Biodentine contrary to other materials where bismuth oxide is preferred as a radiopacifier. The reason for such a preference might be due to some study results which show that zirconium oxide possesses biocompatible characteristics and is indicated as a bioinert material with favorable mechanical properties and resistance to corrosion [27].

Grech et al. [9] in a study evaluating the prototype radiopacified tricalcium silicate cement, Bioaggregate, and Biodentine, concluded that all materials had radiopacity values greater than 3 mm Al. Similar results were obtained by Camilleri et al. [8]. On the other hand, a clinical observation stated that the radiopacity of Biodentine is in the region of dentin and the cement is not adequately visible in the radiograph. This posed difficulty in terms of practical applications [28]. This subjective comment was further supported in a study by Tanalp et al. [29] where the radiopacity of Biodentine was found to be lower compared to other repair materials tested (MM-MTA, and MTA Angelus) and slightly lower than the 3 mm Al baseline value set by ISO. Though these results should be interpreted with caution as experimentation conditions, preservation periods and other factors might affect the results of radiopacity studies, they also indicated that radiopacity quality might need to be further investigated. Another consideration should be made based on the clinical scenarios where Biodentine is intended to be used. In cases where there is direct contact with the surrounding connective tissue, biocompatibility is of primary significance. Though the confirmation of adequate placement of the material is important in such cases by relying on the radiopacity value, one can prefer to make a judgement by clinical observation in case the usage of additives to obtain high radiopacity value is likely to compromise the overall biocompatibility.

### 2.8. Solubility

Grech et al. [9] demonstrated negative solubility values for a prototype cement, Bioaggregate, and Biodentine, in a study assessing the physical properties of the materials. They attributed this result to the deposition of substances such as hydroxyapatite on the material surface when in contact with synthetic tissue fluids. This property is rather favorable as they indicate that the material does not lose particulate matter to result in dimensional instability.

### 2.9. Effect on the Flexural Properties of Dentine

An important issue related to the usage of calcium silicate based materials is their release of calcium hydroxide on surface hydrolysis of their calcium silicate components [3]. On the other hand, it has also been indicated that prolonged contact of root dentine with calcium hydroxide as well as MTA has detrimental and weakening effects on the resistance of root dentine [30, 31]. Therefore, it is critical to consider the effects of released calcium hydroxide on dentine collagen, specifically in procedures where there is a permanent contact of dentine with calcium silicate based materials. Sawyer et al. [32] evaluated whether prolonged contact of dentine with calcium silicate based sealers would have any influence on its mechanical properties. According to the results of their study where they compared Biodentine with MTA Plus, they determined that both materials altered the strength and stiffness of the dentine tissue after aging in 100% humidity. They suggested that though dentine's ability to withstand external impacts and resistance to external forces might not be affected to a critical extent when used in very thin layers such as pulp capping material or as an apical plug, careful consideration is necessary when obturating the entire root canal system with these materials or when using them for the purpose of dentine replacement [32].

### 2.10. Microleakage

When specifically used as a liner or base material, leakage of Biodentine should especially be considered as leakage may result in postoperative sensitivity and secondary caries, leading to the failure of the treatment. Koubi et al. [33] were the first to assess the in vitro marginal integrity of open-sandwich restorations based on aged calcium silicate cement and resin-modified glass ionomer cement. Results of glucose filtration analysis after one-year aging showed that both materials displayed similar leakage patterns and Biodentine performed as well as the resin modified glass ionomer cement. Another significant property of Biodentine was that it did not require specific preparation of the dentin walls. They explained the good marginal integrity of Biodentine with the ability of calcium silicate materials to form hydroxyapatite crystals at the surface. These crystals might have the potential to increase the sealing ability, especially when formed at the interface of the material with dentinal walls. Furthermore, the interaction between the phosphate ions of saliva and the calcium silicate based cements might lead to the formation of apatite deposits, thereby increasing the sealing potential of the material. The authors additionally expressed the nanostructure and small size of the forming gel of the calcium silicate cement as one of the factors that influenced the sealability as this texture allowed the material to better spread onto the surface of the dentine. Slight expansion was also noted for these materials which contributed to their better adaptation [33].

Another study comparing the leakage of Biodentine with a resin modified glass ionomer (Fuji II LC) was one by Raskin et al. [34] where silver penetration was evaluated in cervical lining restorations. Similar results were reported to those by Koubi et al. [33] and Biodentine as a dentine substitute in cervical lining restorations or as a restorative material in approximal cavities, when cervical extent was under CEJ, appeared to perform well without any conditioning. The only disadvantage was related to the operating time that was determined to be longer than the resin modified glass ionomer [33].

A contradictory report was by Camilleri et al. [13] in a study comparing the physical properties of Biodentine with a conventional (Fuji IX) and resin modified glass ionomer (Vitrebond). When used as a dentin replacement material in the sandwich technique overlaid with composite, significant leakage occurred at the dentine to material interface. On the other hand, materials based on glass ionomer cement displayed no chemical and physical changes or microleakage when the materials were used as bases under composite restorations [13]. Though the contradictory statement could be due to the methodology used for the detection of leakage, further studies are warranted to clarify the leakage occurring with calcium silicate based materials.

### 2.11. Discoloration

One study evaluated Biodentine from this perspective where Biodentine, along with 4 different materials, was exposed to different oxygen and light conditions and spectrophotometric analysis was performed at different periods until 5 days [35]. Favorable results were obtained for Portland Cement (PC) and Biodentine and these 2 materials demonstrated color stability over a period of 5 days. Based on their results, the authors suggested that Biodentine could serve as an alternative for use under light-cured restorative materials in areas that are esthetically sensitive [35].

### 2.12. Wash-Out Resistance

Washout of a material is defined as the tendency of freshly prepared cement paste to disintegrate upon early contact with fluids such as blood or other fluids. The results of the available study on these characteristics of Biodentine did not reveal favorable results as the material demonstrated a high washout with every drop used in the methodology [9]. The authors attributed this result to the surfactant effect water soluble polymer added to the material to reduce the water/cement ratio [9].

## 3. Biocompatibility of Biodentine

Biocompatibility of a dental material is a major factor that should be taken into consideration specifically when it is used in pulp capping, perforation repair or as a retrograde filling. During the aforementioned procedures, the material is in direct contact with the connective tissue and has the potential to affect the viability of periradicular and pulpal cells. Cell death under these circumstances occurs due to apoptosis or necrosis [36]. Therefore, it is essential that toxic materials are avoided and materials promoting repair or that are biologically neutral are preferred during procedures in which the material is directly in contact with the surrounding tissue. Though the information accumulated so far regarding the biocompatibility of Biodentine is rather limited, the available data generally is in favor of the material in terms of its lack of cytotoxicity and tissue acceptability. Han and Okiji [37]compared Biodentine and white ProRoot MTA in terms of Ca and Si uptake by adjacent root canal dentine and observed that both materials formed tag-like structures. They observed that dentine element uptake was more prominent for Biodentine than MTA. The same authors [38] in another study also showed the formation of tag-like structures composed of Ca and P-rich and Si-poor materials. They also determined a high Ca release for Biodentine. Laurent et al. [39] were the first to show the promising biological properties of Biodentine on human fibroblast cultures. In another study by Laurent et al. [40] Biodentine was found to significantly increase TGF-B1 secretion from pulp cells. TGF is a growth factor whose role in angiogenesis, recruitment of progenitor cells, cell differentiation, and mineralization has been highlighted in recent research [40].

In a study performed by Zhou et al. [36], where Biodentine was compared with white MTA (ProRoot) and glass ionomer cement (FujiIX) using human fibroblasts, both white MTA and Biodentine were found to be less toxic compared to glass ionomer during the 1- and 7-day observation period. The authors commented that despite the uneven and crystalline surface topography of both Biodentine and MTA compared to the smooth surface texture of the glass ionomer, cell adhesion and growth were determined to be more favorable in the aforementioned materials compared to glass ionomer. They attributed this to the possible leaching of substances from glass ionomer that adversely affect interactions with the material. On the other hand, in a longer incubation period, surviving cells could overcome the cytotoxic effect of glass ionomer [36].

Another study comparing the biocompatibility and gene expression ability of Biodentine and MTA was one by Pérard et al. [41]. Based on the standpoint that three-dimensional (3D) multicellular spheroid cultures are currently considered to be the in vitro model providing the most realistic simulation of the human tissue environment, they performed a biocompatibility investigation using this type of modelling. Biodentine and MTA were determined to modify the proliferation of pulp cell lines. They observed similarity between Biodentine and MTA validating the indication of these 2 materials for direct pulp-capping as suggested by manufacturers [41].

A recently published article focused on the influence of Biodentine from another perspective and assessed the proliferative, migratory, and adhesion effect of different concentrations of the material on human dental pulp stem cells (hDPSCs) obtained from impacted third molars [42]. Results showed increased proliferation of stem cells at 0.2 and 2 mg/mL concentrations while the cellular activity decreased significantly at higher concentration of 20 mg/mL. Biodentine favorably affected healing when placed directly in contact with the pulp by enhancing the proliferation, migration, and adhesion of human dental pulp stem cells, confirming the bioactive and biocompatible characteristics of the material [42].

## 4. Biodentine as a Vital Pulp Treatment Material

When materials' influences are to be evaluated in terms of pulpal response during vital procedures, in vivo study designs are helpful and animal and human teeth are generally preferred to demonstrate the effects of pulp capping agents. These should further be supported by clinical trials to establish a clear picture regarding the general characteristics of the materials. MTA, which is generally considered a gold standard, has been investigated in various human and animal experimental models. On the other hand, studies comparing MTA with Biodentine in terms of vital pulp treatment behavior are rather limited. The first study to demonstrate the induction of effective dentinal repair was the one by Tran et al. [43] where the material was applied directly on mechanically exposed rat pulps. In their study where Biodentine was compared to MTA and calcium hydroxide in terms of reparative dentine bridge formation, they noted that the structure induced by Ca(OH)_2_ contained several cell inclusions, also called tunnel defects as previously reported by Cox et al. in 1996 [44]. These defective regions were regarded as undesirable areas facilitating the migration of the microorganisms towards the pulp and predisposing the tooth to an endodontic infection. On the contrary, the dentine bridge formation induced by Biodentine showed a pattern well-localized at the injury site unlike that caused by calcium hydroxide that exhibited an expanding structure in the pulp chamber. The quality of the formed dentine was also much more favorable compared to calcium hydroxide and an orthodentin organization was noted in which dentine tubules could be clearly visualized. Moreover, cells secreting the structure well exhibited DSP expression as well as osteopontin expression, which are critical regulators of reparative dentine formation [44].

An interesting clinical and histological study performed on molars to be extracted for orthodontic reasons showed that Biodentine had a similar efficacy to MTA in clinical setting and may well be regarded as an alternative for pulp capping procedures. Complete dentinal bridge formation and absence of an inflammatory response were observed as major findings [45].

Pulpotomy is another vital pulp treatment method in which Biodentine is advocated to be used. This method is widely used in pediatric dentistry and involves the amputation of pulp chamber and the placement of a material for the preservation of the radicular pulp tissue's vitality. This methodology is specifically useful and preferred when the coronal pulp tissue is inflamed and a direct pulp capping is not a suitable option. Shayegan et al. [46] performed a study in which they assessed the pulpal response of primary pig teeth against Biodentine when used as a pulp capping as well as a pulpotomy material after 7,28 and 90 days. Their results showed that Biodentine has bioactive properties, encourages hard tissue regeneration, and provoke no signs of moderate or severe pulp inflammation response. They further noted that the material had the ability to maintain a successful marginal integrity due to the formation of hydroxyapatite crystals at the surface which enhances the sealing ability. Due to its superior sealing potential, there is no risk of microleakage which may cause the pulp to become infected or necrotic and jeopardize the success of vital treatment procedures. Another important comment was that the hard tissue formation due to calcium hydroxide was rather a defense response of the pulp against the irritant nature of the material whereas calcium silicate based materials are compatible with the cell recruitment. Furthermore, the necrotic layer caused by calcium hydroxide appeared to be much larger compared to others [46].

Zanini et al. [47] also evaluated the biological effect of Biodentine on murine pulp cells by analysing the expression of several biomolecular markers after culturing OD-21 cells with or without Biodentine. Their results, consistent with other studies, were in favor of Biodentine, which was found to be bioactive due to its ability to increase OD-21 cell proliferation and biomineralization.

Laurent et al. [40] indicated that though the interactions between pulp capping materials and the injured pulp tissue are yet unclear, there is growing evidence on the role of growth factors, with TGF-*β*1 being the most important one. These factors' main role is the signalling of reparative dentinogenesis. In a recently published article, they assessed the reparative dentin synthesis capacity of Biodentine as well as the ability to modulate TGF-*β*1 secretion by pulp cells which has previously shown to be released from dentine by calcium hydroxide [48, 49]. Using an entire human tooth culture model, they showed that, upon application on the exposed pulp, Biodentine had the potential to significantly increase TGF-*β*1 secretion from pulp cells and induce an early form of reparative dentin synthesis [40].

In addition to the aforementioned favorable biological results, supportive statements were made by Marijana et al. [50], who concluded the therapeutic effects of Biodentine after pulp capping in Vietnamese pigs and the resemblance of the pulp reaction to that caused by ProRoot MTA.

## 5. Case Reports Where Biodentine Is Used

A survey of the available literature shows that there are yet few case reports published that include the usage of Biodentine. However, all articles retrieved display the material as a favorable and promising alternative for clinical applications. Villat et al. [51] performed a partial pulpotomy in an immature second right premolar of a 12-year-old patient whom they followed up until 6 months. The authors detected a fast tissue response radiologically evident by the dentine bridge formation and continuation of root development in the short term. Furthermore, no pain or complaints were reported by the patient along the observation period. They commented that increased speed of pulpal response as well as more homogeneous dentine bridge formation render this material a suitable choice compared to calcium hydroxide [51].

One report has been retrieved in which the use of Biodentine has been assessed as a retrograde material [52]. It describes the management of a large periapical lesion associated with the maxillary right central and lateral incisors of a 24-year-old patient who had a history of previous traumatic injury. Following the use of Biodentine as a retrograde material during apical surgery, the patient was followed for a period of 18 months, during which progressive periapical healing was evident [52].

Although case reports are definitely important resources of confirming a material's suitability for clinical usage, it is undeniable that more reliable results can be achieved through randomized long-term clinical trials. Accumulation of data of long-term clinical trials after a prolonged period might lead to gathering of evidence based data; such has been for mineral trioxide aggregate. Though the chemical characteristics and general features of Biodentine are similar, it is clear that a specific number of clinical trials should be conducted before definite conclusions can be drawn. So far, there is one 3-year clinical trial where Biodentine has been used and in which the material has been assessed in terms of various parameters such as marginal adaptation, interproximal contact, surface roughness, and postoperative pain [12]. Biodentine was found to show favorable clinical performance until a period of 6 months though the other test material (Z100) displayed better scores in terms of anatomical form, marginal adaptation, and proximal contact. After a 1-year period, the authors carried on the investigation by the addition of Z-100 over Biodentine by the sandwich technique which resulted in very satisfactory treatment performance. They concluded that Biodentine is a well-tolerated dentine substitute for posterior teeth for up to 6 months during which abrasion is the main degradation process. No discoloration is noted and the material has even yielded superior results compared to Z-100 in terms of this property. In general, Biodentine was advocated to be used under composite in posterior restorations, supporting the major standpoint from which the material was initially developed, in other words as a dentine replacement material [12].

The summarized reports are the only available clinical information published so far and it is presumed that, as more clinical data is released about Biodentine, the clinician will be able to make a more sound and reliable decision regarding its usage.

## 6. Conclusions

Biodentine, a popular and contemporary tricalcium silicate based dentine replacement and repair material, has been evaluated in quite a number of aspects ever since its launching in 2009 (Table 1). The studies are generally in favor of this product in terms of physical and clinical aspects despite a few contradictory reports. Though accumulation of further data is necessary, Biodentine holds promise for clinical dental procedures as a biocompatible and easily handled product with short setting time. As more research is performed regarding this interesting alternative to MTA, we will be provided with more reliable data and more confidently implement Biodentine into routine clinical applications.

## Figures and Tables

**Table 1:** Overview of studies on Biodentine.

PROPERTY	AUTHORS
Composition	Grech et al. 2013 [7], Camilleri et al. 2012 [4], Camilleri et al. 2013 [8]
Setting time	Grech et al. 2013 [9], Villat et al. 2010 [11]
Compressive strength	Kayahan et al. 2013 [2], Grech et al. 2013 [9]
Microhardness	Grech et al. 2013 [9], Camilleri et al. 2013 [13]
Bond strength	Odabaş et al. 2013 [14], Aggarwal et al. 2013 [15], El-Ma'aita et al. 2013 [16], Hashem et al. 2014 [17], Guneser et al. 2013 [18]
Porosity and material-dentine interface	Atmeh et al. 2012 [23], Camilleri et al. 2013 [20], De Souza et al. 2013 [21], Gjorgievska et al.2013 [22]
Radiopacity	Grech et al. 2013 [9], Camilleri et al. 2013 [8], Tanalp et al. 2013 [29]
Solubility	Grech et al. 2013 [9]
Flexural properties	Sawyer et al. 2012 [32]
Microleakage	Koubi et al. 2012 [33], Raskin et al. 2012 [34], Camilleri et al. 2013 [13]
Discoloration	Vall$\overset{´}{\text{e}}$s et al. 2013 [35]
Wash-out resistance	Grech et al. 2013 [9]
Biocompatibility and Bioactivity	Laurent et al. 2008 [39], Laurent et al. 2012 [40], Han and Okiji 2011 [37], Han and Okiji 2013 [38], Zhou et al. 2013 [36], P$\overset{´}{\text{e}}$rard et al 2013 [41], Luo et al. 2014 [42], Camilleri et al. 2013 [8]
Vital pulp treatment	Shayegan et al. 2012 [46], Zanini et al. 2012 [47], Laurent et al. 2012 [40], Tran 2012 [43], Nowicka et al. 2013 [45], Marijana et al. 2013 [50]
Case reports and clinical studies	Villat et al. 2013 [51], Pawar et al. 2013 [52], Koubi et al. 2013 [12]
